# Nineteen-year forgotten ureteral stent removed under local anaesthetic from a transplanted kidney

**DOI:** 10.1308/rcsann.2024.0066

**Published:** 2024-09-24

**Authors:** SS Gosein, JA Forster, JF Bolton

**Affiliations:** Bradford Teaching Hospitals NHS Foundation Trust, UK

**Keywords:** Retained stent, Forgotten, Local anaesthetic, Flexible cystoscopy, Renal transplant, Stent register

## Abstract

Following renal transplant, ureteral stents aim to minimise ureteroneocystostomy anastomotic complications. Although there is no specified timing for stent removal after transplantation, these are ideally removed at between 2 and 4 weeks. However, forgotten stents can adversely affect renal allograft function and contribute to obstructive uropathy. We present a 59-year-old man with a retained ureteral stent for more than 19 years with an absence of encrustations, fragmentation, migration and stone formation. To our knowledge, this is the longest retained ureteral stent in a renal transplant patient and the first forgotten stent removed via flexible cystoscopy under local anaesthetic.

## Background

Ureteral stents are commonly used to drain the upper and lower urinary tracts. These stents play a major role in many endourological procedures. The first modern ureteral stent was described in the 1960s by Zimskind *et al*.^1^ Over the years there has been medical evolution and progression resulting in various stents. The quest continues for the ideal stent that is affordable, inert and able to inhibit encrustations, migration and fragmentation. Currently, polymeric ureteral double J stents are most commonly used in both prophylactic and therapeutic cases. Prophylactic indications include usage before endoscopic ureteric or renal calculus surgery, reconstructive surgery, non-urological procedures involving ureteral dissection and renal transplantation.^2^ Ureteral stenting during renal transplantation should assist a watertight ureteroneocystostomy, minimising urinary leakage and ureteric stenosis.^3^ Although ureteral stents are beneficial they have potential deleterious complications. These include stent migration, bladder symptoms, encrustations, pain, ureteral stent obstruction and forgotten stents. Delayed removal of forgotten ureteral stents requires carefully planned endoscopic surgery. We present an unusual case of the removal of an ureteral stent forgotten for more than 19 years in a renal transplant patient. This was removed via flexible cystoscopy under local anaesthetic in the absence of encrustations, blockage and migration.

## Case history

**Figure 1:**
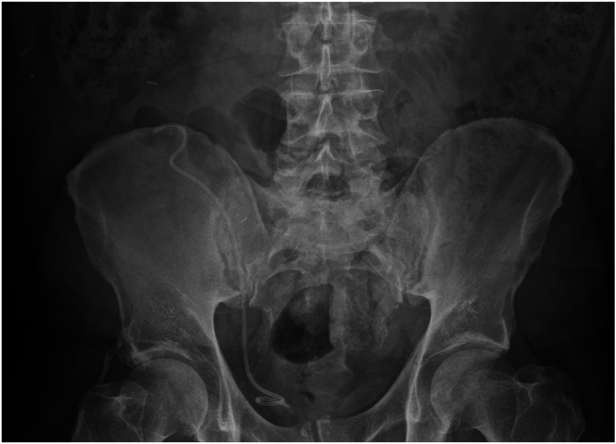
Pelvic radiograph

**Figure 2:**
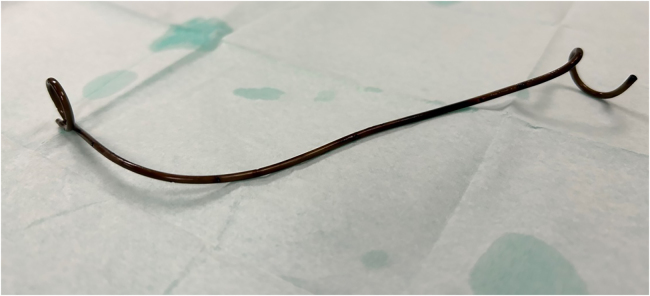
Double J ureteral stent without encrustations

A 59-year-old man with known primary type 1 hyperoxaluria underwent liver and renal transplantation in 2004. The patient has been on immunosuppression with tacrolimus and prednisolone since then. He had routine follow-up in the nephrology clinic for 19 years with no major complaints. He remained well in the absence of urinary tract infections and stent symptoms. His kidney functions fluctuated around his baseline creatinine 120–125μmol/L and estimated glomerular filtration rate 54 to 60. After 19 asymptomatic years, the patient presented to his local hospital with visible haematuria. Flexible cystoscopy was performed that revealed a right ureteral stent. He was then referred for an abdominal x-ray, which showed surgical clips from the previous renal transplant and a correctly sited, retained double J ureteric stent without radio-opaque encrustations or stone formation (Figure 1). A decision was made to assess the distal coil of the stent with flexible cystoscopy; if no encrustations were seen, then a gentle attempt at removal under local anaesthetic would be performed. Flexible cystoscopy and stent removal under local anaesthetic was performed without any complications (Figure 2). The patient was discharged to the nephrology clinic on the same day and had no complications in the 6 months following the procedure.

## Discussion

Ureteral stents play a major role in the urological armamentarium. These stents are used in endourological procedures during stone treatments, reconstructive surgery of the upper urinary tract, decompression of the collecting system and in renal transplant surgery. In renal transplants, ureteral stenting is done prophylactically to prevent vesicoureteric complications such as urine leaks, ureteric stenosis and obstruction.^3^ A meta-analysis evaluated five randomised, controlled clinical trials and 44 case series of routine stenting following renal transplantation. In the clinical controlled trials, the urological complication rate following routine stenting was 1.5% compared with 9% without stenting.^4^

Despite the multitude of beneficial roles in renal transplants, ureteral stents can cause severe complications that can ultimately contribute to patients' morbidity and mortality. If forgotten, ureteral stents can cause urinary tract infections, haematuria, stent migration, blockage and non-function, encrustations, stone formation and difficulty in removal. Although there is no defined period for stent removal, most centres favour a shorter length of time, ranging between 2 and 4 weeks to avoid complications associated with longer stent dwelling times.^2^ To avoid forgotten ureteral stents and their associated complications, various stent registers have been implemented in which an electronic alert indicates the timeframe when the ureteral stent is scheduled for removal.

Currently, there is no universally used national stent register within the United Kingdom. Local registries vary within National Health Service trusts. This poses a complex problem that must be solved by individual urology, transplant, oncology and radiology departments within hospitals. Failure to accurately record, monitor and remove stents can lead to undesirable litigation and ultimately increased morbidity. The British Association of Urological Surgeons recommended concise discharge information including date of insertion, expected date of removal and a clear communication portal among patient, urology department and general practitioners. This is found in the patient information leaflet *Living with a Stent*.^5^ A uniformly used reliable and accessible register would benefit urologists and other specialties that deploy stents including transplant, oncology, radiology and gynaecology. However, it is recognised that this is no easy task and robust IT, information governance, expenditure and human resources would be required.

Ureteral stents have evolved over the years and their materials have a great influence on their efficacy. Their properties should minimise associated stent complications such as encrustations, bacterial adhesions, patient discomfort and flexibility. Polymeric stents are the most commonly used stent because of their low cost and inert properties, and are usually well tolerated by transplant patients. One of the daunting challenges faced is ureteral stent encrustations. Encrustations have been related to the indwelling time and stent indications (stone vs non-stone). According to the severity of encrustations, additional and complicated procedures are necessary for removal. As stated by Legrand *et al*, the encrustation rate for non-stone indications was 1.3% at <4 months, 5.2% at <6 months and increased to 10% for long-term indications.^6^ This is not evident in this case report, making it unusual and rare, especially in the absence of other stent complications affecting renal allograft function. Even though this patient has a medical history of type 1 primary hyperoxaluria, the absence of encrustations can be attributed to the liver and renal transplant, which corrected the metabolic effect. Lack of encrustations enabled this forgotten stent to be safely removed and well tolerated under local anaesthetic in the endoscopy suite.

## Conclusions

A 19-year-old forgotten transplant ureteral stent without encrustations is rare, especially without the development of obstructive uropathy, migration and fragmentation. The role of a robust stent register is of paramount importance to prevent such occurrences and a national registry may be favourable in the near future for transplant and other stents.
